# Drug Design and Development for Rare Hematologic Diseases

**DOI:** 10.3390/ph16101469

**Published:** 2023-10-16

**Authors:** Bruno Fattizzo, Marco Capecchi, Irene Motta

**Affiliations:** 1 SC Ematologia, Fondazione IRCCS Ca’ Granda Ospedale Maggiore Policlinico, 20122 Milan, Italy; 2 Department of Oncology and Hemato-Oncology, University of Milan, 20122 Milan, Italy; 3 Angelo Bianchi Bonomi Hemophilia and Thrombosis Center, Fondazione IRCCS Ca’ Granda Ospedale Maggiore Policlinico, 20122 Milan, Italy; 4 Division of Hematology, Clinica Moncucco, 6900 Lugano, Switzerland; 5 Department of Clinical Sciences and Community Health, University of Milan, 20122 Milan, Italy; 6 Unit of Medicine and Metabolic Diseases, Fondazione IRCCS Ca’ Granda Ospedale Maggiore Policlinico, 20122 Milan, Italy

The last decade has seen an exponential increase in therapeutic options for rare hematologic diseases. The latter encompass benign conditions, including congenital anemias, autoimmune cytopenias, bone marrow failure syndromes (i.e., aplastic anemia and paroxysmal nocturnal hemoglobinuria), and rare hemostatic disorders, as well as neoplastic ones (i.e., low-risk myelodysplastic syndromes and systemic mastocytosis) [1,2,3,4,5,6]. On the whole, these disorders often represent a diagnostic challenge given their rarity, but also due to their heterogeneous clinical phenotype. Once diagnosed, many of them are treated with supportive therapies only (i.e., blood and plasma transfusions, plasmapheresis, etc.) for many years, representing a true unmet clinical need. Novel treatment, including oral small molecules, intravenous or subcutaneous monoclonal antibodies, small interfering RNAs, gene therapy, and many others have the potential to change the natural history of these disorders [1,2,3,4,5,6]. In this Special Issue of Pharmaceuticals, experts in rare congenital and acquired hematologic disorders have addressed the above-mentioned topics, giving evidence-based and personal insights on this exciting evolving scenario.

Regarding congenital disorders, Bou-Fakhredin and colleagues [7] lead us in the world of hemoglobin F (HbF) induction in beta-thalassemia and sickle cell disease, discussing the use of lentiviral and genome-editing strategies and their limitations. They focus on the pharmacologic agents for HbF induction that have been developed, starting from a deep understanding of globin regulation. The role of the hematopoietic niche in hemoglobinopathies is further addressed by Aprile et al. [8], who discuss the recent findings highlighting multiple alterations of the marrow microenvironment and their functional implications. In particular, they describe the possible role of targeting the bone marrow niche by ameliorating the quality of patient-derived stem cells to improve the effect of gene therapy. Finally, Scaramellini et al. [9] refresh the concept of iron homeostasis and its broad implications in congenital and acquired forms of iron-related disorders.

Switching to acquired autoimmune forms, Mingot-Castellano and colleagues [10] recapitulate unmet clinical needs in immune thrombocytopenias and provide a list of possible remedies through the advent of novel therapies, including next-generation thrombopoietin receptor agonists, fostamatinib, and neonatal Fc receptor inhibitors. Beyond immune thrombocytopenia, Bortolotti et al. [11] discuss how the oral thrombopoietin receptor agonist, eltrombopag, that has revolutionized the treatment of acquired aplastic anemia, may be beneficial in several neglected off-label settings. The Authors focus on the use of eltrombopag in post-hematopoietic stem cell transplant aplastic anemia and poor graft function, providing insights on the immunomodulating properties of the drug. Regarding transplant, Ardissino and colleagues [12] address the use of complement inhibitors in transplant-related thrombotic microangiopathy, a rare and often under-recognized condition with a high fatality rate.

In the field of hemostatic disorders, Gualtierotti et al. [13] provide an update on novel drugs for hemophilia patients based on thrombin generation restoring or coagulation factor VIII mimicking. Beyond the already approved bispecific monoclonal antibody emicizumab, they discuss novel non-replacement drugs designed to reduce the treatment burden of patients with hemophilia A or B with or without inhibitors.

Finally, Sciumè and colleagues [14] face the challenging field of systemic mastocytosis, a heterogeneous condition with multi-organ involvement. They highlight that the disease has now two target therapies approved, midostaurin and avapritinib, based on the understanding of the typical molecular alteration of *cKIT* gene.

Overall, the novel therapies discussed in this Special Issue are at different phases of development (either preclinical or clinical) and will likely be positioned at different disease stages or in specific settings soon. Along with efficacy, which may vary according to disease phenotype, new toxicities are emerging and novel specific monitoring and preventive strategies will have to be developed. Finally, each novel drug poses a challenge to the treating physician, who will have to individualize treatment by choosing the best therapy for the best patient.
